# Neonatal Hemodynamic Characteristics of the Recipient Twin of Twin-To-Twin Transfusion Syndrome Not Treated with Fetoscopic Laser Surgery

**DOI:** 10.3390/children9111766

**Published:** 2022-11-17

**Authors:** Edouard Chambon, Taymme Hachem, Elodie Salvador, Virginie Rigourd, Claire Bellanger, Julien Stirnemann, Elsa Kermorvant-Duchemin, Pierre Tissieres, Yves Ville, Alexandre Lapillonne

**Affiliations:** 1 Neonatal Intensive Care Unit, APHP Necker-Enfants Malades University Hospital, 75015 Paris, France; 2 Department of Obstetrics and Fetal Medicine, APHP Necker-Enfants Malades University Hospital, 75015 Paris, France; 3 UFR de médecine, Université Paris Cité, Site Cordeliers, 75006 Paris, France; 4 Pediatric Intensive Care Unit, APHP Bicêtre University Hospital, 94270 Le Kremlin-Bicêtre, France; 5 UFR de médecine, Université de Paris Saclay, 94270 Le Kremlin-Bicêtre, France

**Keywords:** recipient twin, twin-to-twin transfusion syndrome, hemodynamics, cardiac failure

## Abstract

Background: This paper’s intent is to describe the neonatal hemodynamic characteristics of recipient twins of monochorionic pregnancies complicated with twin-to-twin transfusion syndrome (TTTS), born without prenatal fetoscopic selective laser coagulation (FSLC). Methods: Retrospective analysis of hemodynamic characteristics was performed during the first five days of life of recipient twins from untreated TTTS. Results: Forty-two recipient twins were included and divided into three groups: no hemodynamic impairment (NoHI) (*n* = 15, 36%), isolated high blood pressure (HighBP) (*n* = 12, 28%), and cardiac failure group (CF) (*n* = 15, 36%). Patients of both CF and HighBP groups had high systolic blood pressure during the first 12 h of life and ventricular hypertrophy at early echocardiography. Cardiac failure occurred at a median age of 14 h (IQR = 6–24) and was followed by a drop in systolic and diastolic blood pressure. Acute kidney injury was more frequent (93% vs. 25%, *p* < 0.001) and severe (*p* <0.001) in the CF group than in the HighBP group. The mortality rate in the CF group was 40%. Factors associated with CF were twin anemia-polycythemia sequence (*p* = 0.012), very preterm birth (*p* = 0.040), and polycythemia (*p* = 0.002). Conclusion: One-third of recipient twins born without prenatal FSLC developed life-threatening cardiac failure during the first 24 h of life.

## 1. Introduction

Twin-to-twin transfusion syndrome (TTTS) is an unbalanced transfer of volemia through placental intertwin anastomoses and occurs in 10 to 20% of monochorionic diamniotic pregnancies, with a fetal mortality approaching 90% and a morbidity in survivors of approximately 50% if left untreated [1,2]. TTTS is characterized by oliguric oligohydramnios in one twin (the donor), and polyuric polyhydramnios in the co-twin (the recipient) [1,3]. The effects of TTTS on cardiac function in the recipient fetus have been well described [4,5]. They are characterized by increased systemic vascular resistance, elevated blood pressure, and subsequent fetal cardiomyopathy leading to progressive cardiac failure [6,7]. Whenever possible, prenatal intrauterine fetoscopic selective laser coagulation (FSLC) of the anastomotic placental vessels is the best first-line treatment for TTTS to alleviate the cardiovascular and hemodynamic burden caused by the disease [4,8,9]. However, some cases cannot benefit from FSLC because of spontaneous delivery prior to intrauterine surgery or when the disease occurs in the late third trimester when laser treatment is no longer technically feasible [3].

Due to the hemodynamic imbalance and associated prematurity, neonates with TTTS not treated with FSLC are at high risk for neonatal mortality, neonatal morbidity including necrotizing enterocolitis, severe brain injury, polycythemia, and long term neurodevelopmental delay [10,11,12,13]. However, the immediate postnatal cardiac and hemodynamic impairments in untreated born recipients are poorly described and remain a challenge for neonatologists. The aim of this monocentric cohort study is to describe the postnatal hemodynamic characteristics of the recipient twins after untreated TTTS and to identify the risk factors associated with severe hemodynamic impairments.

## 2. Materials and Methods

### 2.1. Population

A monocentric cohort study was conducted over a 9-year period in a single referral tertiary medical center for high-risk pregnancies and TTTS. All consecutive cases of newborns with untreated TTTS admitted to the neonatal intensive care unit (NICU) between 1 January 2011 and 31 December 2019 were considered for the study. Inclusion criteria were: inborn recipient twin from a multiple pregnancy with TTTS (isolated or associated with Twin Anemia Polycythemia Sequence (TAPS) [14] or intrauterine growth restriction) not treated with FSLC and admitted alive in the neonatal intensive care unit. Exclusion criteria were: donor twin, uncertainty regarding classification as donor or recipient, pregnancy with isolated TAPS, prenatal intervention by FSLC, prematurity before 24 weeks, early-onset neonatal bacterial infection, infants with distributive, and hypovolemic or obstructive shock from other causes.

### 2.2. Data Collection

Clinical and biological data were collected retrospectively from medical records using a case report file with predefined parameters that combined prenatal, perinatal, and neonatal relevant information. Postnatal data were collected for each case until the fifth day of life or death.

Prenatal data included gestational age estimated by first trimester ultrasound, Quintero staging of TTTS [15], intrauterine fetal death, and any prenatal intervention such as amnioreduction or selective termination during pregnancy. Acidosis at birth was defined by an arterial pH < 7.20 in the umbilical cord [16]. APGAR-score was classified as low if <7 at 1 or 5 min [17]. TAPS was diagnosed by the association of polycythemia (hemoglobin level at birth >20 g/dL) in one twin and anemia (Hb < 11 g/dL) in the co-twin [18]. Percentiles of weight were calculated using the AUDIPOG curves [19]. Infants were classified as extremely, very, moderately, and late preterm if gestational age was <28 weeks, 28 to 31 + 6 weeks, 32 to 33 + 6 weeks, and 34 to 36 + 6 weeks, respectively [20]. Blood pressure was measured noninvasively with a fitted cuff size. High blood pressure was defined by a systolic blood pressure equal to or above the 95th percentile for gestational age [21]. Systolic, diastolic, and mean blood pressures were expressed as medians and quartiles by period of 6 h. 

Postnatal cardiac ultrasound assessment included diastolic interventricular septum diameter (IVSd) and left ventricular systolic function in the parasternal long axis view (M-mode measurements of left ventricular end-diastolic and end-systolic diameters with thickness of the septal and posterior walls) [22]. Ventricular hypertrophy was defined as a ventricular septum larger than 3 mm in the end-diastolic time in preterm infants or 4 mm in term infants [23]. Left ventricular systolic dysfunction was defined as a left ventricular ejection fraction below 55% or a left ventricular shortening fraction below 30% [22]. When detailed echocardiographic measurements were not available, a statement of ventricular hypertrophy or impaired shortening fraction in the medical record was used to define ventricular hypertrophy or left ventricular dysfunction, respectively.

Urine output was measured by weighing diapers every 3 h and expressed as median and quartiles by a period of 6 h. Acute kidney injury (AKI) was defined according to the neonatal modified KDIGO definition (Kidney Diseases: Improving Global Outcomes) [24]. International definitions for necrotizing enterocolitis and bronchopulmonary dysplasia were used [25].

Medications included antihypertensive treatment, fluid expansion, and inotropic support. Inotropic support was defined by any inotrope drug administered during the first 5 days of life (dobutamine or milrinone regardless of dose and duration of treatment).

### 2.3. Management Protocol

The protocol for the management of recipient twins was as follows. An initial echocardiographic assessment was performed as soon as possible after admission (preferably within the first two hours of life) and repeated regularly depending on the hemodynamic status of the infants. Heart rate, blood pressure, and urine output were recorded every 3 h. Hemodilution by partial exchange transfusion was indicated if the hematocrit at birth was above 65%. Nicardipine was used for high systolic blood pressure in combination with ventricular hypertrophy on echocardiography. Dobutamine was used as the first-line treatment for left ventricular dysfunction at echocardiography. Milrinone was used in place of dobutamine when the left ventricular dysfunction persisted despite treatment with nicardipine and dobutamine. Cautious fluid expansion with saline solution (10 mL/kg) was performed when echocardiography showed evidence of preload dependence (i.e., respiratory fluctuations in aortic blood flow peak velocity [26]). Blood tests, including blood gasses, blood counts, and serum electrolytes, were performed at the discretion of the physicians.

### 2.4. Statistics

Quantitative values were expressed as median and interquartile range and compared using the Mann–Whitney test. Qualitative variables were expressed as percentages and compared using Fischer’s exact test. Ordinal qualitative variables were compared using the Kruskall–Wallis test. A value of *p* < 0.05 was considered significant.

## 3. Results

### 3.1. Study Population and Clinical Characteristics

**Figure 1:**
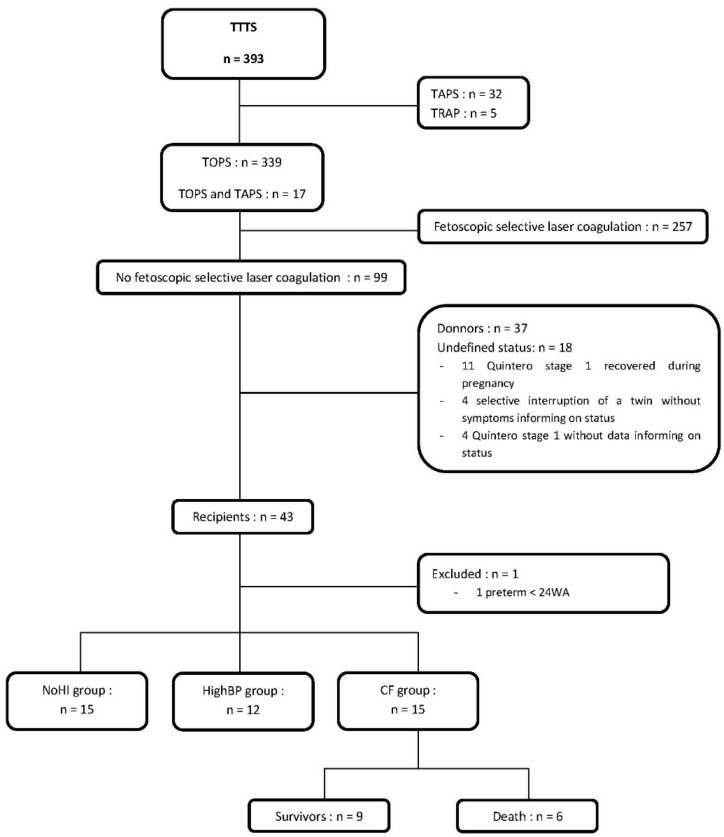
Flow chart of the study.

During the 9-year study period, 393 newborns with TTTS were born at our institution. Of the 43 recipient twins who had not benefited from FSLC and were considered for the study, 42 were included (Figure 1). The study population was divided into 3 groups based on hemodynamic characteristics: (i) 15 cases had no hemodynamic impairment (i.e., normal blood pressure and no cardiac failure, NoHI group); (ii) 12 cases had isolated high blood pressure without other hemodynamic impairment (HighBP group); (iii) 15 cases presented cardiac failure, defined as left ventricular systolic dysfunction (CF group) (Table 1).

### 3.2. Characterization of the Hemodynamic Disorders in the Recipient Twins

The NoHI group had no hemodynamic disorder. Their clinical characteristics differed from those of the two other groups by a higher rate of prenatal death of the donor twin, a higher gestational age at birth, and a higher birth weight (Table 1).

**Figure 2:**
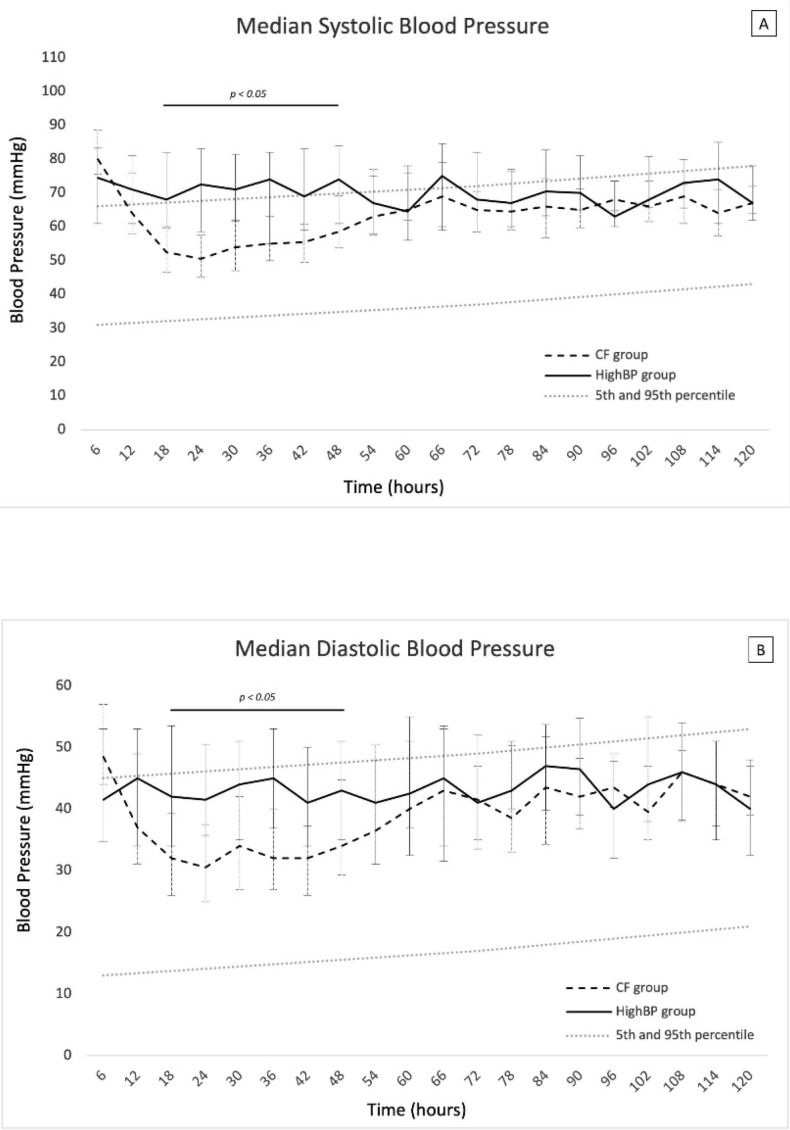
Systolic (**A**) and diastolic (**B**) blood pressure of recipient twins with cardiac failure (CF group) and with isolated high systolic blood pressure (HighBP group). All data are presented as median values with 1st and 3rd quartiles.

Comparison of the other two groups (i.e., HighBP and CF groups) showed that they had similar clinical characteristics at birth (gestational age, birth weight, blood pressure, ventricular hypertrophy) (Table 2). During the first 12 h of life, systolic blood pressure was higher than the 95th percentile in both groups, and diastolic blood pressure was within the normal range (Figure 2). High blood pressure was treated with nicardipine in both groups, with no significant difference between the two groups in age at onset and maximal nicardipine dose (Table 2). Ventricular hypertrophy was also observed in both groups but occurred significantly earlier in the CF group than in the HighBP group (100% at day 0 versus 60%, respectively; *p* = 0.023) (Supplementary Figure S1). The median age at onset of left ventricular dysfunction in the CF group was 14 h of life [6,7,8,9,10,11,12,13,14,15,16,17,18,19,20,21,22,23,24]. One patient (7%) in the CF group did not receive any inotropic treatment and recovered with antihypertensive treatment only. The other patients in the CF group were treated with milrinone (7%), dobutamine (60%), or both (27%). Fluid expansion was used more frequently in the CF than the HighBP group (53% vs. 8%, respectively, *p* = 0.019).

Between 18 and 48 h of life, systolic and diastolic blood pressure values were significantly lower in the CF group than in the HighBP group. During this period, systolic and diastolic blood pressure in the CF group were approximately at the mean and at the 30th percentile of normal values for gestational age, respectively. 

**Figure 3:**
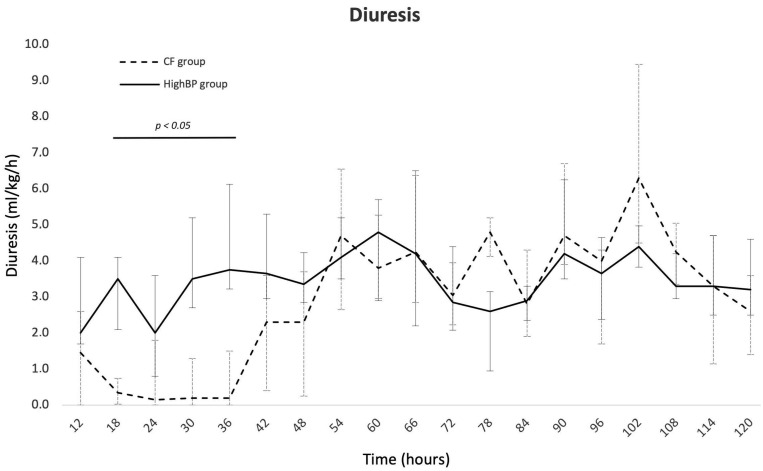
Urine output of recipient twins with cardiac failure (CF group) and with high systolic blood pressure alone (HighBP group). Data are presented as median values with 1st and 3rd quartiles.

AKI was more frequent in the CF group than in the HighBP group (93% vs. 25%; *p* < 0.001) and also more severe (grade 3 = 87% vs. 0%, *p* < 0.001). Urine output was similar in both groups at 12 h of life but decreased to less than 1 mL/kg/h between 18 and 36 h of life in the CF group, whereas it remained above 2 to 3 mL/kg/h in the HighBP group (Figure 3). Blood urea nitrogen and serum creatinine increased significantly in the CF group and were significantly higher than in the HighBP group between day 1 and day 4 and at day 1 and 2, respectively (Table 2, Supplementary Figure S2).

### 3.3. Factors Associated with Cardiac Failure (Table 3)

Search for associated factors of CF was performed by comparing the characteristics of the CF group with those of the NoHI and the HighBP groups combined (Table 3). The factors associated with CF were the presence of an associated TAPS, found only in the CF group (*p* = 0.012), emergency cesarean section for fetal rescue (*p* = 0.020), very preterm birth (*p* = 0.040), low APGAR score at 1 and 5 min (*p* = 0.020), polycythemia (*p* = 0.002), and hematocrit greater than 65% (*p* = 0.047). All infants with hematocrit upper than 65% received hemodilution.

**Table 3:** Risk factors associated with cardiac failure (CF) in recipient twins. * No possibility of calculating Odds Ratio.

	NoHI + HighBP		CF		OR	95% CI	p
	n = 27		n = 15				
**Pregnancy**							
Threatened preterm labor, n (%)	6	(22)	2	(13)	0.6	(0.1–3.4)	1.00
Prenatal death of the donor twin							
Gestational diabetes, n (%)	1	(4)	1	(7)	1.9	(0.1–32.1)	1.00
Pre-eclampsia, n (%)	1	(4)	0	(0)	*		1.00
Premature rupture of membranes, n (%)	4	(15)	0	(0)	*		0.28
Antenatal corticosteroid therapy, n (%)	19	(70)	10	(67)	0.8	(0.2–3.3)	1.00
**TTTs**							
Amnioreduction, n (%)	14	(52)	8	(53)	1.1	(0.3–3.8)	1.00
Gestational age, median (Q1–Q3)	28	(26–29)	28	(28–29)			0.65
Associated-TAPS, n (%)	0	(0)	4	(27)	*		**0.012**
Quintero stage							
Quintero stage 1 or 2, n (%)	16	(59)	6	(40)	0.5	(0.1–1.7)	0.34
Quintero stage 3 or 4, n (%)	8	(30)	9	(60)	3.6	(0.9–13.4)	0.52
Quintero stage 5, n (%)	3	(11)	0	(0)	*		0.54
Selective interruption during pregnancy, n (%)	6	(22)	0	(0)	*		0.07
**Birth**							
Mode of delivery							
Elective cesarean section, n (%)	20	(74)	5	(33)	0.2	(0.1–0.7)	**0.020**
Emergency cesarean section, n (%)	7	(26)	10	(67)	5.7	(1.4–22.6)	
Term							
Very preterm, n (%)	14	(52)	13	(87)	6.0	(1.1–32.1)	**0.040**
Moderate or late preterm, n(%)	13	(48)	2	(13)	0.2	(0–0.9)	
Male, n (%)	12	(44)	7	(47)	1.1	(0.3–3.9)	1.00
Weight discordance with the twin > 25%, n (%)	7	(26)	3	(20)	0.7	(0.2–3.3)	1.00
APGAR score							
Low 1-min APGAR score, n (%)	9	(33)	12	(80)	8.0	(1.8–35.8)	**0.009**
Low 5-min APGAR score, n (%)	3	(11)	7	(47)	7.0	(1.5–33.7)	**0.020**
Umbilical cord acidosis, n (%)	0	(0)	3	(20)	*		**0.040**
Hemoglobin at birth > 20 g/dL, n (%)	2	(7)	8	(53)	14.3	(2.5–83.2)	**0.002**
Hematocrit at birth > 65%, n (%)	1	(4)	4	(27)	9.5	(1.0–94.4)	**0.047**

### 3.4. Mortality Rate

None of the patients in both the NoHI group and the HighBP group died. In contrast, 6 (40%) of the CF patients died during the first 5 days of life, all because of severe cardiac failure. The median time of death was 47 h (IQR = 26–65). There was no significant difference in clinical characteristics, blood pressure profiles, urine output, or biological indices between the CF infants who died and those who survived (Supplementary Table S1, Supplementary Figures S3 and S4). In contrast, those who died had earlier left ventricular dysfunction (*p* = 0.042, Supplementary Figure S5) and were treated earlier with nicardipine (*p* = 0.03), received a higher dose of dobutamine (*p* = 0.006), and were treated more frequently with both milrinone and dobutamine (*p* = 0.01) (Supplementary Table S1).

## 4. Discussion

By describing three groups of recipient twins born after a pregnancy with TTTS based on their hemodynamic characteristics, we clarify the data from the literature that seem to be contradictory. Indeed, some authors did not find a significant higher risk of high blood pressure in recipients, nor did they observed higher levels of endothelin in the cord blood [27]. For others, there is no doubt about the reality of systemic hypertension in recipients, this hypertension being associated with biventricular myocardial hypertrophy [28].

In our study, we described, in a sufficiently large cohort of patients, that there are three different postnatal hemodynamic outcomes, each of them concerning roughly one-third of the recipients not treated by FSLC. One of the groups, born less premature, exhibited neither hypertension nor hemodynamic failure, likely reflecting a less active TTTS. In this group, half of them were born from a pregnancy characterized by the prenatal death of the donor twin (selective termination or spontaneous intrauterine fetal death), suggesting that the loss of the co-twin had cured the TTTS.

The other two groups had significant hemodynamic disorders characterized by high systolic blood pressure and ventricular hypertrophy, consistent with fetal descriptions. During pregnancy with TTTS not treated by FSLC, the recipient fetus suffers from an increased preload with an absolute increase in circulating volume and cardiac output [4]. Increased afterload due to higher systemic arterial pressure (presence of vasoactive substances and consequence of inappropriate renin–angiotensin stimulation due to transfer from donor) leads to left ventricular hypertrophy [4,6,7]. Taken together, these mechanisms are responsible for the development of cardiomyopathy with diastolic and systolic dysfunction [4].

One-third of the infants developed postnatal cardiac failure. The first hours of life of the infants in this group are characterized by systolic hypertension and ventricular hypertrophy, as was also observed in the HighBP group. Interestingly, only subtle early clinical differences existed between the HighBP and CF groups. They had similar high systolic blood pressure, antihypertensive treatment, and ventricular hypertrophy, but the latter developed earlier in the CF group. The CF group subsequently developed a ventricular dysfunction leading to a drop in both systolic and diastolic blood pressure and to a consequent acute renal injury. This relative hypotension raises the question of the blood pressure target in this population. Blood pressure may need to be maintained at the 95th percentile, and nicardipine dose reduced if progression to hypotension occurs. Short-term renal dysfunction has been described in both laser and non-laser patients [9], but the elevated serum creatinine and blood urea nitrogen levels associated with the decrease in urine output are more likely explained by the low systemic blood flow and renal perfusion rate caused by the cardiac failure. Despite early antihypertensive treatment and high doses of inotropes, mortality was observed in 40% of the patients in the CF group.

Identification of risk factors for postnatal cardiac failure in recipient twins has never been reported. We found that TAPS and polycythemia were associated with hemodynamic failure. Whether hyper-viscosity or the severity of exchange between twins, resulting in higher circulating volume, is responsible for the reduction in myocardial compliance remains unclear. Surprisingly, but in agreement with the literature, we found no association between the severity of TTTS, as determined by Quintero grading and the risk of cardiac failure. This may be because Quintero grading only partially quantifies cardiac involvement and functional cardiovascular changes [29].

We acknowledge that our study has several limitations. The retrospective design has its own limitations. The ultrasound measurements were performed by the physicians on duty with a lack of standardization and which were not uniformly recorded in the medical records; however, distributive, hypovolemic and obstructive shocks from other causes were systematically excluded. We did not measure biochemical markers such as natriuretic peptide, endothelin, and troponin to better investigate cardiac involvement and damage.

In conclusion, three groups of recipient twins born after a pregnancy with TTTS not treated by FSLC are described on the basis of their hemodynamic characteristics. Approximately one-third of them developed life-threatening cardiac failure within the first 24 h of life. Associated TAPS and polycythemia are risk factors for cardiac failure. Forty percent of patients with cardiac failure died within the first five days of life, despite early antihypertensive treatment and high doses of inotropes. Close clinical and echocardiographic hemodynamic monitoring should be initiated immediately after birth in these high-risk infants. Additional postnatal studies with biological markers are needed to better explore and predict the postnatal hemodynamic outcomes.

## Figures and Tables

**Table 1:** General characteristics of the population divided into three groups according to the hemodynamic status during the first five days of life: no hemodynamic impairment (NoHI group), isolated high blood pressure (HighBP group), and cardiac failure (CF group). Data are presented as median values and 1st and 3rd quartiles or *n* (%). * Outcomes calculated in survivors.

	NoHI		HighBP		CF	
	*n* = 15		*n* = 12		*n* = 15	
**Pregnancy**						
Threatened preterm labor, *n* (%)	3	(20)	3	(25)	2	(13)
Prenatal death of the donor twin, *n* (%)	8	(53)	1	(8)	0	(0)
Gestational diabetes, *n* (%)	0	(0)	1	(8)	1	(7)
Pre-eclampsia, *n* (%)	0	(0)	1	(8)	0	(0)
Premature rupture of membranes, *n* (%)	2	(13)	2	(17)	0	(0)
Antenatal corticosteroid therapy, *n* (%)	9	(60)	10	(83)	10	(67)
**Birth**						
Gestational age, median (Q1–Q3)	33	(32–34)	29	(28–30)	29	(28–31)
Male, *n* (%)	5	(33)	7	(58)	7	(47)
Weight (g), median (Q1–Q3)	1800	(1665–2082)	1293	(1168–1533)	1265	(1150–1607)
Weight (percentile), median (Q1–Q3)	53	(31–77)	71	(55–79)	66	(34–75)
% difference of weight with the twin, median (Q1–Q3)	20.1	(8.7–28)	22.8	(11.8–30.1)	18.2	(12.9–22.8)
APGAR score						
1 min, median (Q1–Q3)	8	(8–9)	6	(4–8)	5	(2–6)
5 min, median (Q1–Q3)	10	(9–10)	9	(8–9)	8	(4–9)
Umbilical cord pH, median (Q1–Q3)	7.36	(7.31–7.39)	7.37	(7.36–7.39)	7.30	(7.26–7.37)
**Hemodynamic morbidity**						
High systolic blood pressure, *n* (%)	0	(0)	12	(100)	15	(100)
Ventricular hypertrophy, *n* (%)	2/11	(33)	12	(100)	15	(100)
Patent ductus arteriosus requiring treatment, *n* (%)	0	(0)	0	(0)	1	(7)
**Other neonatal morbidities**						
Hemodilution, *n* (%)	0	(0)	1	(8)	4/15	(27)
Bronchopulmonary dysplasia, *n* (%) *	0	(0)	2	(17)	1/9	(11)
Severe intraventricular hemorrhage (>2), *n* (%)	0	(0)	1	(8)	3/15	(20)
Periventricular leukomalacia, *n* (%) *	0	(0)	1	(8)	4/9	(44)
Secondary sepsis, *n* (%) *	2	(13)	2	(17)	4/9	(44)
Necrotizing enterocolitis, *n* (%) *	1	(7)	1	(8)	0/9	(0)
Acute kidney injury, *n* (%)	0	(0)	3	(25)	14/15	(93)
**Death**						
Death in the first five days of life, *n* (%)	0	(0)	0	(0)	6	(40)
Age at death (hours), median (Q1–Q3)					47	(26–65)

**Table 2:** Comparison of hemodynamic characteristics between the high blood pressure (HighBP) group and the cardiac failure (CF) group. IVSd: Interventricular septum diameter.

	HighBP		CF		p
	n = 12		n = 15		
**Birth**					
Gestational age, median (Q1–Q3)	29	(28–30)	29	(28–31)	0.77
Weight (g), median (Q1–Q3)	1293	(1168–1533)	1265	(1150–1607)	0.98
Weight (percentile), median (Q1–Q3)	71	(55–79)	66	(34–75)	0.26
% difference of weight with the twin, median (Q1–Q3)	22.8	(11.8–30.1)	18.2	(12.9–22.8)	0.51
APGAR score					
1 min, median (Q1–Q3)	6	(4–8)	5	(2–6)	0.35
5 min, median (Q1–Q3)	9	(8,9)	8	(4–9)	0.15
Umbilical cord pH, median (Q1–Q3)	7.37	(7.36–7.39)	7.30	(7.26–7.37)	0.05
**Hemodynamic characteristics**					
High systolic blood pressure, n (%)	12	(100)	15	(100)	1.00
Ventricular hypertrophy, n (%)	12	(100)	15	(100)	1.00
Maximum IVSd (mm), median (Q1–Q3)	4.2	(3.8–5.1)	5.0	(4.2–5.3)	0.32
Hydrocortisone hemisuccinate, n (%)	2	(17)	6	(40)	0.41
Renal-dose dopamine, n (%)	0	(0)	4	(27)	0.10
Fluid expension with saline solution, n (%)	1	(8)	8	(53)	**0.019**
**Antihypertensive treatment**					
Nicardipine, n (%)	12	(100)	15	(100)	1.00
Starting time (hours of life), median (Q1–Q3)	4	(4–23)	3	(2–16)	0.18
Maximal dose (μg/kg/min), median (Q1–Q3)	0.6	(0.5–1.2)	1.0	(0.7–1.8)	0.08
**Inotropic treatment (Milrinone, Dobutamine), n (%)**	0	(0)	14	(93)	
Milrinone, n (%)			5	(33)	
Starting time (hours of life), median (Q1–Q3)			22	(16–39)	
Maximal dose (μg/kg/min), median (Q1–Q3)			0.2	(0.2–0.2)	
Dobutamine, n (%)			13	(87)	
Starting time (hours of life), median (Q1–Q3)			13	(6–26)	
Maximal dose (μg/kg/min), median (Q1–Q3)			20.0	(15.0–20.0)	
**Biological parameters**					
pH in the first 2 days of life, median (Q1–Q3)	7.33	(7.29–7.37)	7.26	(7.23–7.29)	**<0.001**
Lactate in the first 2 days of life, median (Q1–Q3)	1.6	(1.2–2.2)	2.1	(1.5–3.3)	0.07
Acute kidney injury, n (%)	3	(25)	14	(93)	**<0.001**
Stage 1 of KDIGO classification, n (%)	2	(17)	1	(7)	0.57
Stage 2 of KDIGO classification, n (%)	1	(8)	0	(0)	0.44
Stage 3 of KDIGO classification, n (%)	0	(0)	13	(87)	**<0.001**
Maximum serum blood urea nitrogen (mmol/L), median (Q1–Q3)	6.7	(6.1–8.0)	13.3	(8.8–17.9)	**0.003**
Maximum serum creatinine (µmol/L), median (Q1–Q3)	77.0	(62.3–87.5)	134.0	(90.8–157.8)	**0.005**
Urinary examinations, n (%)	9	(75)	5	(33)	
Urinary sodium excretion (mmol/L), median (Q1–Q3)	38.0	(33.0–54.0)	28.5	(13.0–36.0)	**0.011**
Proteinuria (g/L), median (Q1–Q3)	0.15	(0.08–0.24)	0.30	(0.18–0.49)	**0.032**
Urinary to serum ratio of urea nitrogen, median (Q1–Q3)	8.34	(7.08–8.70)	5.74	(3.28–6.51)	**0.017**
Urinary to serum ratio of creatinine, median (Q1–Q3)	10.74	(9.24–13.17)	11.92	(6.30–19.34)	0.44
Urinary sodium to potassium excretion ratio, median (Q1–Q3)	5.17	(3.26–8.65)	1.44	(0.99–1.62)	**<0.001**

## Data Availability

All data generated or analyzed during this study are included in this article and its supplementary material files. Further enquiries can be directed to the corresponding author.

## Supplementary Materials

The following supporting information can be downloaded at: https://www.mdpi.com/article/10.3390/children9111766/s1, Figure S1: Percentage of recipient twins with left ventricular hypertrophy depending on postnatal age (days) in the cardiac failure (CF) group and in the high systolic blood pressure (HighBP) group; Figure S2: Serum concentration of blood urea nitrogen (**A**) and creatinine (**B**) during the first 5 days of life recipient twin with cardiac failure (CF group) and with high systolic blood pressure (HighBP group). Data are presented as median values and 1st and 3rd quartiles; Figure S3: Systolic (**A**) and diastolic (**B**) blood pressure of the recipients of the cardiac failure (CF) group according to survival. All data are presented as median values and 1st and 3rd quartiles; Figure S4: Urine output of the recipients of the cardiac failure (CF) group according to survival. All data are presented as median values and 1st and 3rd quartiles; Figure S5: Kaplan-Meyer graphic of the time without left ventricular dysfunction in recipient twins with cardiac failure who died or who survived; Table S1: Comparison of the infants who died with those who survived in the cardiac failure (CF) group.
