# Supplementary material for: Neonatal Hemodynamic Characteristics of the Recipient Twin of Twin-To-Twin Transfusion Syndrome Not Treated with Fetoscopic Laser Surgery
Source: Children (Basel). 2022 Nov 17;9(11):1766. doi: 10.3390/children9111766 (PMC9689049; doi:10.3390/children9111766)
Supplement: Supplementary file 1 [file children-09-01766-s001.zip › children-2026301-supplementary.pdf]

Supplementary materials

Table S1. Comparison of the infants who died with those who survived in the cardiac failure (CF) group.

	DEATH n = 6		SURVIVORS n = 9		*p*
**Pregnancy**					
Threatened preterm labor, n	1	(0)	1	(0)	*1.00*
Gestational diabetes, n (%)	0	(0)	1	(11)	*1.00*
Pre-eclampsia, n (%)	0	(0)	0	(0)	*1.00*
Premature rupture of membranes, n (%)	0	(0)	0	(0)	*1.00*
Antenatal corticosteroid therapy, n (%)	3	(50)	7	(78)	*0.33*
**TTTs**					
Amniodrainage, n (%)	4	(67)	4	(44)	*0.60*
Gestational age, median [Q1 - Q3]	29	[28 - 30]	28	[27 -28]	*0.46*
Associated-TAPS, n (%)	2	(33)	2	(22)	*1.00*
Quintero stage					
Quintero stage 1 or 2, n (%)	2	(33)	4	(44)	*1.00*
Quintero stage 3 or 4, n (%)	4	(67)	5	(55)	
**Birth**					
Mode of delivery					
Elective cesarean section, n (%)	1	(16)	4	(44)	*0.58*
Emergency cesarean section, n (%)	5	(83)	5	(55)	
Gestational age, median [Q1 - Q3]	29	[28 - 30]	29	[29 - 31]	*0.28*
Male, n (%)	3	(50)	4	(44)	*1.00*
Weight (g), median [Q1 - Q3]	1458	[1024 - 1654]	1240	[1200 - 1445]	*1.00*
Weight (percentile), mediane [Q1 - Q3]	75	[43 - 83]	58	[36 - 68]	*0.22*
Difference of weight with the twin in %, median [Q1 - Q3]	21.2	[14.7 - 24.9]	18.2	[12.1 - 19.3]	*0.61*
APGAR score					
M1, median [Q1 - Q3]	6	[2 - 6]	4	[2 - 6]	*1.00*
M5, median [Q1 - Q3]	7	[4 - 8]	8	[5 - 9]	*1.00*
Umbilical cord pH, median [Q1 - Q3]	7.30	[7.19 - 7.30]	7.32	[7.29 - 7.38]	*0.51*
**Neonatal morbidities**					
Hemoglobin at birth (g/dl), median [Q1 - Q3]	19.3	[17.6 - 23.5]	20.0	[14.8 - 22.1]	*0.61*
Hematocrit at birth (%), median [Q1 - Q3]	55.8	[50.9 - 66.7]	59.5	[45.8 - 62.0]	*0.61*
Acute kidney injury, n (%)	6	(100)	8	(89)	*1.00*
Fluid bolus with saline solution, n (%)	4	(67)	4	(44)	*0.61*
Age at first fluid bolus, median [Q1 - Q3]	17	[15 - 18]	24	[19 - 27]	*0.34*
Number of fluid boluses, median [Q1 - Q3]	2	[0 - 3]	0	[0 - 1]	*0.22*
**Antihypertensive treatment**					
Nicardipine, n (%)	6	(100)	9	(100)	*0.40*
Age at onset (hours of life), median [Q1 - Q3]	2	[2 - 3]	4	[3 - 22]	***0.03***
Maximal dose (µg/kg/min), median [Q1 - Q3]	1.5	[1.0 - 2.0]	0.8	[0.7 - 1.5]	*0.69*
**Inotropic treatment (Milrinone, Dubutamine)**					
Both milrinone and dobutamine treatments, n (%)	4	(67)	0	(0)	***0.01***
Milrinone, n (%)	4	(67)	1	(11)	*0.09*
Start time (hours of life), median [Q1 - Q3]	19	[16 - 28]	39		
Maximal dose (µg/kg/min), median [Q1 - Q3]	0.2	[0.2 - 0.3]	0.2		
Dobutamine, n (%)	6	(100)	7	(78)	*0.49*
Start time (hours of life), median [Q1 - Q3]	6	[5 - 11]	19	[14 - 31]	*0.06*
Maximal dose (µg/kg/min), median [Q1 - Q3]	20.0	[20.0 - 20.0]	15.0	[10.0 - 15.0]	***0.006***

<u>Figure S1.</u> Percentage of recipient twins with left ventricular hypertrophy depending on postnatal age (days) in the cardiac failure (CF) group and in the high systolic blood pressure (HighBP) group.

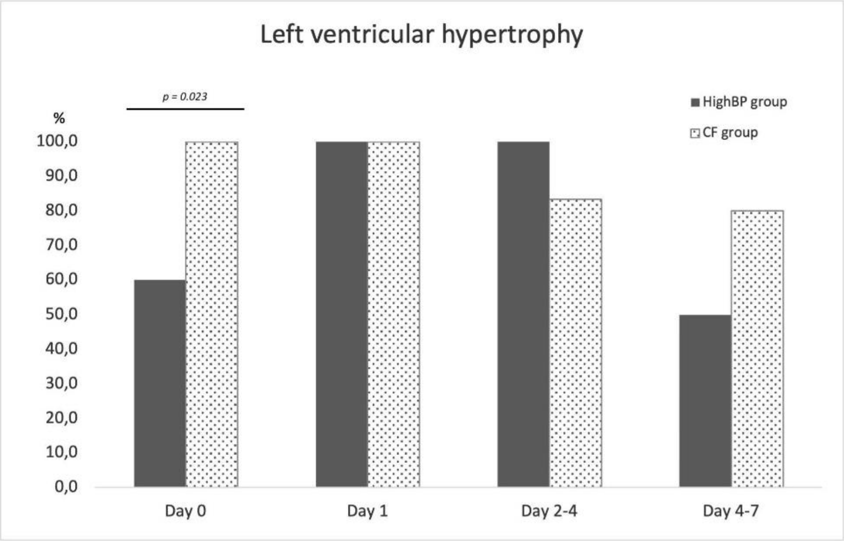

<u>Figure S2</u>. Serum concentration of blood urea nitrogen (A) and creatinine (B) during the first 5 days of life recipient twin with cardiac failure (CF group) and with high systolic blood pressure (HighBP group). Data are presented as median values and 1st and 3rd quartiles.

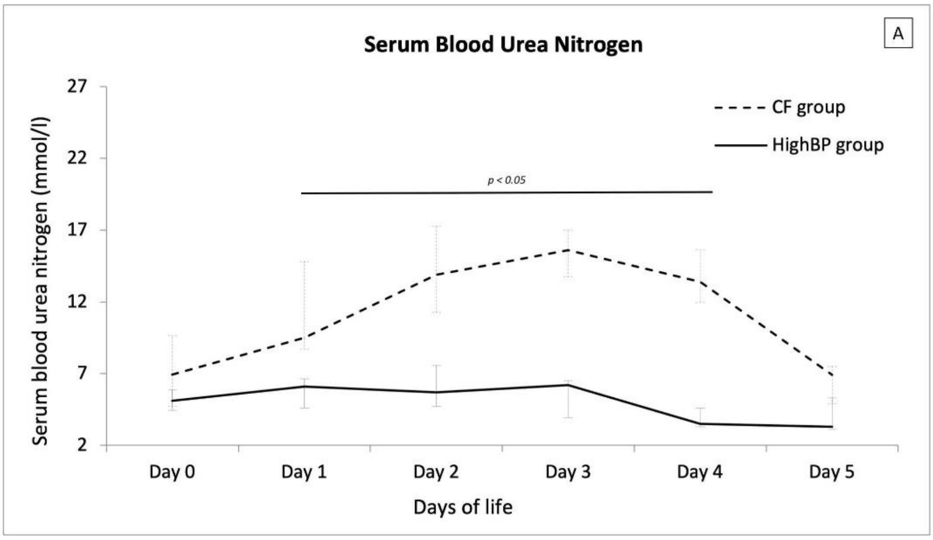

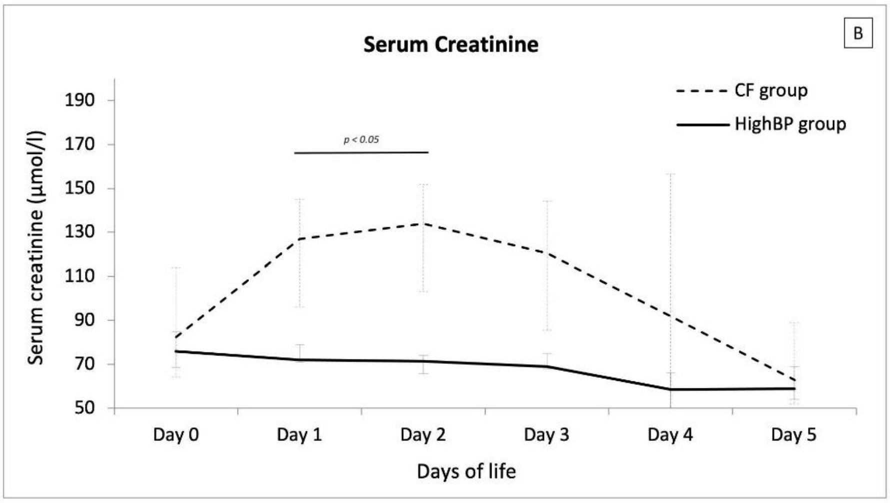

Figure S3. Systolic (A) and diastolic (B) blood pressure of the recipients of the cardiac failure (CF) group according to survival. All data are presented as median values and 1st and 3rd quartiles.

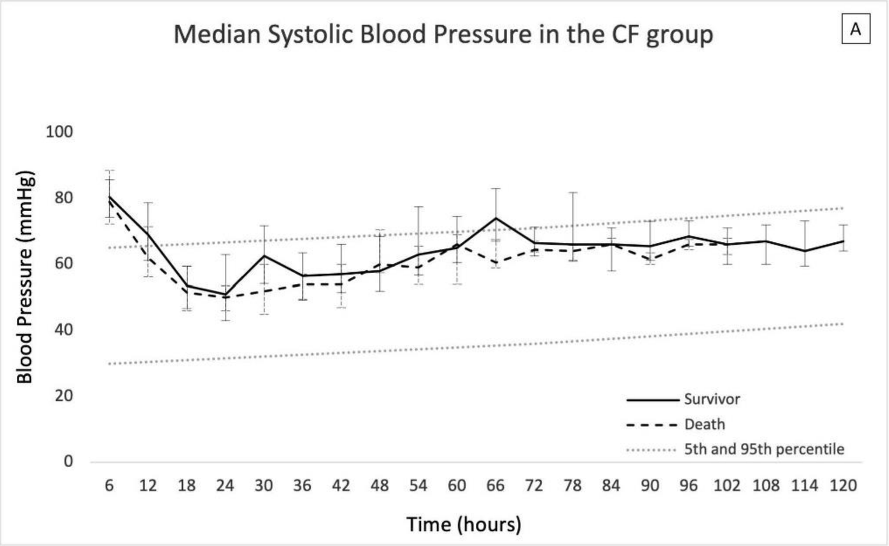

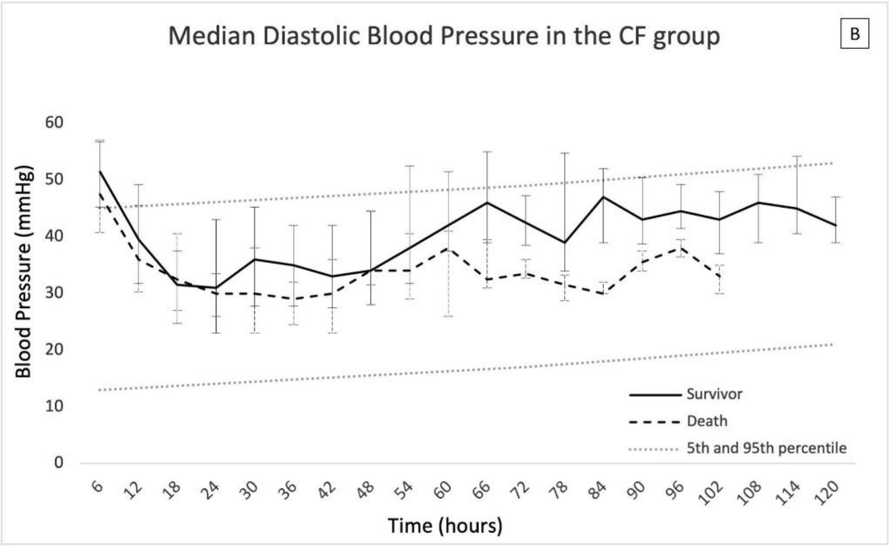

Figure S4. Urine output of the recipients of the cardiac failure (CF) group according to survival. All data are presented as median values and 1st and 3rd quartiles.

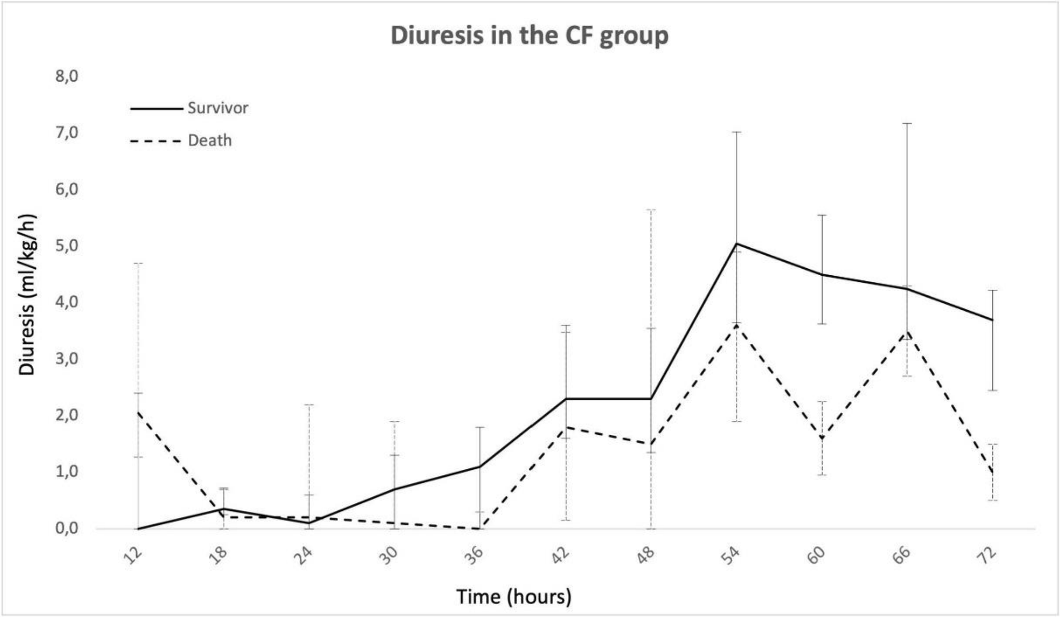

Figure S5. Kaplan-Meyer graphic of the time without left ventricular dysfunction in recipient twins with cardiac failure who died or who survived.

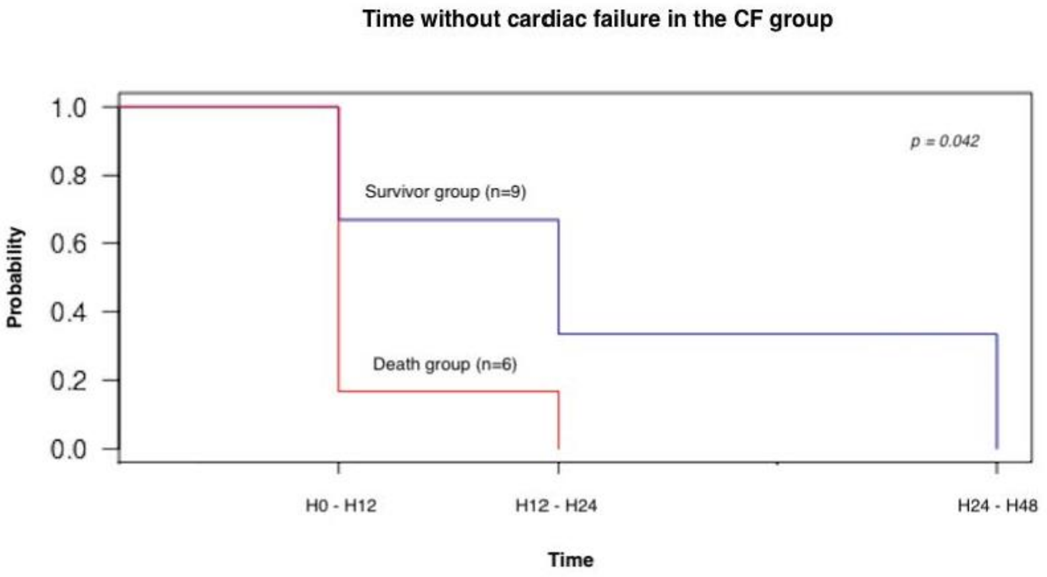
Time without cardiac failure in the CF group
Probability
1.0
0.8
0.6
0.4
0.2
0.0
p = 0.042
Survivor group (n=9)
Death group (n=6)
H0 - H12
H12 - H24
H24 - H48
Time
